# METTL3/LINC00662/miR-186-5p feedback loop regulates docetaxel resistance in triple negative breast cancer

**DOI:** 10.1038/s41598-022-20477-0

**Published:** 2022-10-06

**Authors:** Lei Jing, Liu Lan, Zhang Mingxin, Zhang Zhaofeng

**Affiliations:** 1 Department of Surgical Oncology, Baoji Maternal and Child Health Hospital, Baoji, 721000 Shaanxi China; 2 Department of Gastroenterology, The First Affiliated Hospital of Xi’an Medical University, Xi’an, 710082 Shaanxi China

**Keywords:** Breast cancer, Cell growth

## Abstract

Insight into the mechanism of docetaxel resistance in breast cancer may help to improve prognosis. We aimed to investigate the role of N6-methyladenosine (m6A) and the METTL3/LINC00662/miR-186-5p pathway in regulating docetaxel resistance in triple negative breast cancer (TNBC). We have recruited 193 pathologically diagnosed TNBC patients from 2016 to 2017 in our hospital. Quantitative real-time PCR was used to evaluate the expression of LINC00662 and miR-186-5p both in vivo and in vitro. CCK8 tests were used to assess cell viability. ELISA was used for protein expression evaluation. Dual luciferase reporter gene assay and RNA pull-down were used to evaluate the interaction between LINC00662 and miR-186-5p. m6A levels were enhanced in breast cancer tissues and cells. LINC00662, miR-186-5p and METTL3 were differentially expressed in vivo, and METTL3 expression was associated with LINC00662 and miR-186-5p expression. LINC00662 and miR-186-5p were differentially expressed in vitro; LINC00662 promoted cell viability and decreased the apoptosis rate, whereas miR-186-5p inhibited cell viability and increased the apoptosis rate. Furthermore, we found that METTL3 regulated m6A levels in docetaxel-resistant breast cancer cells by regulating the expression of LINC00662. Moreover, LINC00662 and miR-186-5p regulated the cell viability rate of docetaxel-resistant breast cancer cells. Further experiments showed that LINC00662 directly interacted with miR-186-5p to exert biological functions; besides miR-186-5p could regulate the expression of METTL3. METTL3 promotes m6A levels and docetaxel resistance in breast cancer by regulating the expression of LINC00662 and miR-186-5p; more experiments are needed to clarify the role of m6A regulation in drug resistance.

## Introduction

Breast cancer is the most commonly diagnosed cancer in both developed and less-developed countries, and its incidence is continually increasing in the developing world^1^. Triple negative breast cancer is the most malignant pathological type of breast cancer, a large number of patients were diagnosed in the late stages, which leads to poor prognosis^2^. AC-T chemotherapy, combining doxorubicin (A), cyclophosphamide (C), and paclitaxel (T), is the predominant treatment for breast cancer, especially for patients with triple-negative breast cancer (TNBC)^3^. However, a majority of TNBC experience chemotherapy resistance^4^, for example almost 60% of TNBC patients present with local recurrence and distant metastasis after AC-T chemotherapy^5^. In this context, we will further investigate the mechanism of AC-T chemotherapy resistance in TNBC.

Recently, it was shown that m6A (N6-methyladenosine) majorly induced drug resistance through epigenetically regulate drug resistant candidate gene^6^. m6A “writers”, including METTL3 (methyltransferase-like 3), METTL14, and WTAP (Wilms’ tumor 1-associating protein), have methyltransferase activity and methylate adenosine residues at the N(6) position of RNAs^7,8^. The m6A process plays important parts in mRNA stability, destabilization, and degradation to regulate target genes and mediate biological functions, including drug resistance^9^. For example, m6A methylation of mRNAs can encode naive pluripotency-promoting transcripts and promote differentiation of ESCs^7^. Ban et al. found that an ontogenetic long non-coding RNA (lncRNA)-KBCAROD was stabilized by m6A methylation in head and neck squamous cell carcinoma (HNSCC) to promote cancer progression^10^. Wu et al. found that lncRNA RP11 was induced by m6A and triggered the dissemination of colorectal cancer cells by upregulation of Zeb1 protein expression^11^. It was reported that m6A contributes to cancer cell proliferation, self-renewal, and resistance to radiotherapy and chemotherapy as well^9,12^.

In this manuscript, we found that m6A level was associated with docetaxel resistance of TNBC; besides, m6A writer METTL3 was found to induce docetaxel resistance. Here, we have shown the METTL3/LINC00662/miR-186-5p feedback loop to induce METTL3 expression upon docetaxel resistance.

## Material and methods

### Patients

We recruited 193 pathologically diagnosed triple negative breast cancer patients from 2016 to 2017.

All patients underwent surgical treatment and postoperative review, specifically imaging tests (X-ray/MRI) and blood tests (Routine Blood Test/Tumor markers like CEA、CA153) were performed For all postoperative patients, once every 3 months in the first year, once every 6 months in the next 2 years, and once every year in the last 2 years. All patients were informed about the risks and benefits (The benefit is that free post-operative review can be provided to clarify gene expression and typing. The risk is that frequent follow-up and contact are required) of taking part in our study and provided written consent. Our researchers have confirmed that informed consent was obtained from all the recruited patients. This study met the requirement of the Declaration of Helsinki and was approved by the Ethics Committee of the First Affiliated Hospital of Xi’an Medical University.

### Cell culture and transfection

Breast cancer cell line MDA-MB-231 was purchased from the American Type Culture Collection (Manassas, VA, USA). MDA-MB-231 cells were cultured in 90% L-15 (GIBCO, 41300039) supplemented with 10% fetal bovine serum (FBS) in 5% CO_2_ at 37 °C. MDA-MB-231 were transfected with LINC00662, METTL3, and miR-186-5p up- and downregulated lentiviruses (GeneChem, Shanghai, China) according to the manufacturer’s instructions. MOI for the lentiviruses was 10, except for miR-186-6p and METTL3 up regulated lentiviruses, of which was 20. Enhanced Infection Solution purchased from GeneChem (Shanghai, China) was used to assist the transfection.

### Generation of docetaxel-resistant MDA-MB-231 cell line

Parental cells (MDA-MB-231) were treated with 1 µg/mL docetaxel for 48 h followed by docetaxel-free medium for 48 h. This process was repeated until cells could stably survive in 1 µg/mL docetaxel medium. Then we increased the concentration of docetaxel to 2, 4, 6, and 8 µg/mL. Finally, MDA-MB-231 cells that could stably survive in 8 µg/mL docetaxel were regarded as docetaxel-resistant cells.The docetaxel-resistant MDA-MB-231 cell line was named MDA-MB-231-DR.

### Quantitative real-time PCR (qRT-PCR)

Total RNA was extracted from breast cancer tissue and adjacent non-diseased tissues and cell lines using TRIzol reagent (Thermo Fisher Scientific). Breast cancer tissues and adjacent non-diseased tissues were freshly frozen. Adjacent non-diseased tissues refers to more than 5 cm away from the edge of cancer tissue. Then ReverTra Ace (Toyobo Co., Ltd., Osaka, Japan) was used for cDNA reverse transcription. qRT-PCR was carried out using Thunderbird SYBR qPCR Mix (Toyobo Co., Ltd., Osaka, Japan) and a LightCycler 2.0 (Roche Molecular Systems, Inc., Pleasanton, CA, USA). LINC00662 and miR-186-5p levels were measured and normalized using the GADPH and U6 control. Supplementary Table S1 lists the primers used in this study. The results were analyzed using the 2^−ΔΔCt^ method.

### Apoptosis

The FITC Annexin V Apoptosis Detection Kit I (BD Pharmingen TM, New Jersey, USA) was used for apoptosis testing. Target cells were stained with Annexin V–FITC at room temperature for 15 min, followed by flow cytometry to detect fluorescence intensity.

### Cell viability

Cell viability was evaluated using CCK‑8 assays. Cells were resuspended and plated in a 96‑well plate at a concentration of 1 × 10^3^/well. In the docetaxel treatment group, 1 mg/mL docetaxel was added to the cells, followed by incubation for 24 h. After culturing for 24, 48, 72, 96, and 120 h, respectively, 10 µL CCK‑8 was added to each well and incubated for 2 h. Phosphate-buffered saline was used as a blank control. The absorbance was detected at 450 nm.

### ELISA

The ELISA assays were performed on tissues. The tissue blocks were minced and placed in pre-cooled PBS (0.02 mol/L, pH 7.0–7.2) for washing. Then, the tissue block was moved into a glass homogenizer, and 5–10 ml of pre-cooled PBS was added to fully homogenize the obtained homogenate. The prepared homogenate was centrifuged at 5000 × *g* for 5 min, and the supernatant was collected to detect the expression of METTL3, METTL14, FTO, and ALKBH5 by an ELISA kit (R&D Systems, USA). The absorption was measured at 450 nm, with a correction wavelength of 540 nm.

### m6A

An EpiQuik m6A RNA Methylation Quantification Kit (Colorimetric) was used to determine m6A levels in target cells and tissues according to the manufacturer’s instructions (total RNA is the substrate). At least 200 ng RNA was used for each m6A test sample.

### Dual luciferase reporter gene assay

The pGL3-LINC00662 promoter (Promega, MA, USA) was transfected into the MDA-MB-231 cell lines using the pcDNA3.1 and pcDNA3.1 vector and sh-NC. The wild-type (WT) and mutant (Mut) binding sites of the LINC00662 sequence were cloned into the pmirGLO luciferase vector (Promega) to construct LINC00662-WT and LINC00662-Mut, which were then co-transfected with miR-186-5p mimics or NC mimics, respectively, into the MDA-MB-231 cell line. Luciferase activity was detected using the Dual-Luciferase Reporter Assay System (Promega).

### RNA pull-down

The miR-186-5p-WT, miR-186-5p-Mut, and NC constructs were synthesized and biotin-labeled by GeneCreate (Wuhan, China). After being treated with RNase-free DNase I (Roche) and purified by a RNeasy Mini Kit (Qiagen, Valencia, CA, USA), biotin-labeled sense or antisense oligos of LINC00662 were incubated with 1 mg of whole-cell lysates from target cells for 1 h at 25 °C. Next, the complexes were isolated by streptavidin agarose beads (Invitrogen). The RNA present in the pull-down material was measured by qRT/PCR.

### RNA immunoprecipitation (RIP)

pSL-MS2-12X, pSL-MS2-LINC00662 and pSL-MS2-LINC-mut was cotransfected with pMS2-GFP (Addgene) into target cells using Lipofectamine 3000. Forty-eight hours later, Target cells were lysed and incubated with IgG, m6A and METTL3 antibody for immunoprecipitation. The enriched RNA was measured by qRT/PCR.

### Statistics

GraphPad Prism 8.2.1 (La Jolla, CA, USA) was used for statistical analysis and data visualization. Student’s t-test and one-way analysis of variance were used to test the differences among groups. P < 0.05 was considered to indicate statistical significance. All experiments were repeated three times.

## Results

### LINC00662 expression profile and m6A levels in breast cancer patients

**Figure 1:**
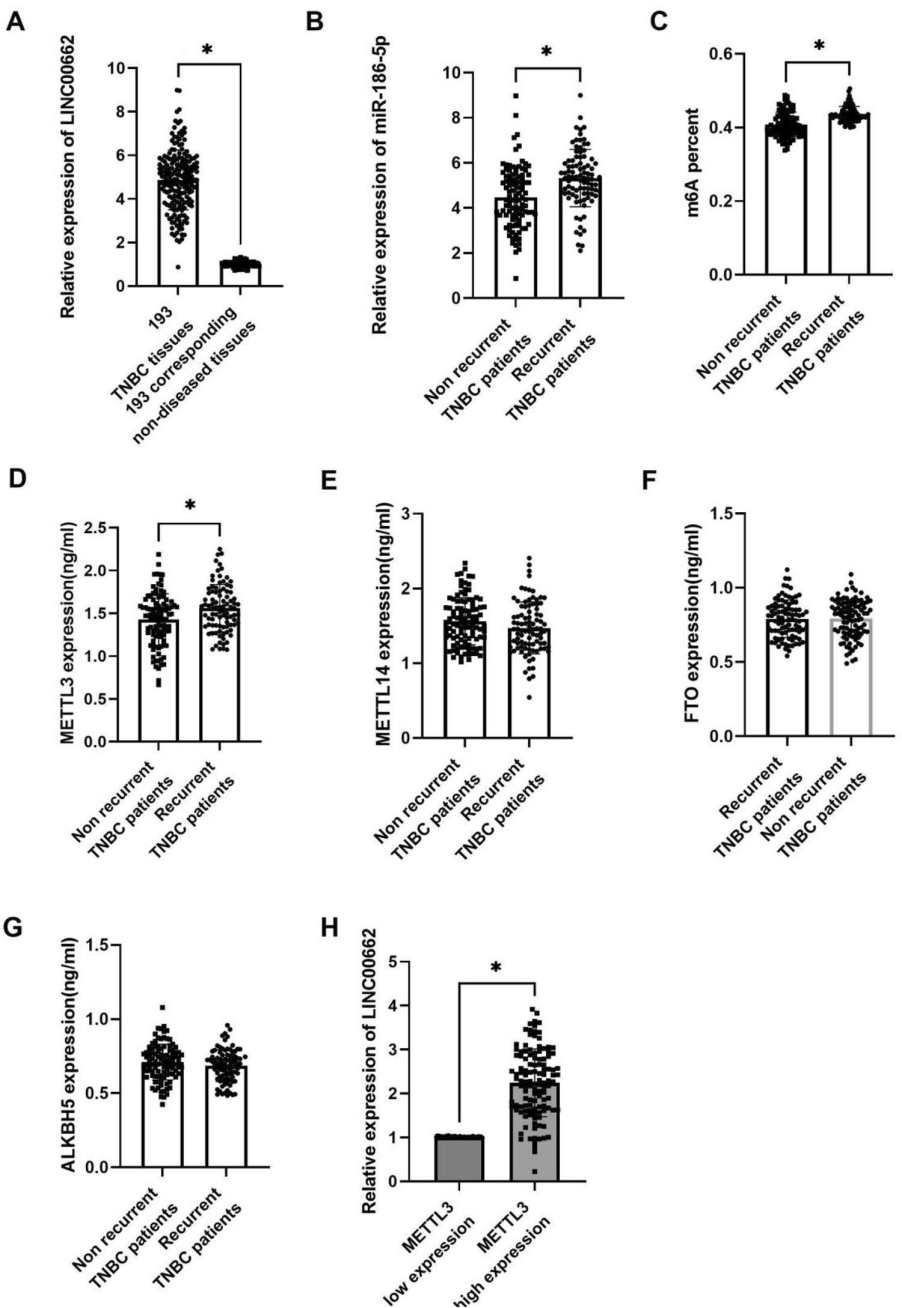
LINC00662 expressed higher in TNBC tissues and recurrent patients. The expression of m6A and METTL3 were upregulated in recurrent TNBC tissues. However, METTL14, FTO and ALKBH5 were not significantly differentially expressed. (**A**) The expression of LINC00662 in 193 TNBC patients compared with their adjacent non-diseased tissues by qRT-PCR; (**B**) the expression of LINC00662 in recurrent and non-recurrent TNBC patients by qRT-PCR; (**C**) the m6A level in recurrent and non-recurrent TNBC patients; (**D**) the expression of METTL3 in recurrent and non-recurrent TNBC patients by ELISA; (**E**) the expression of METTL14 in recurrent and non-recurrent TNBC patients by ELISA; (**F**) the expression of FTO in TNBC patients by ELISA; G The expression of ALKBH5 in TNBC patients by ELISA; H LINC00662 expression in METTL3 low and high expressed TNBC patients by qRT-PCR. The mean METTL3 expression is 1.6 ng/mL, which was used to determine the low and high expression of METTL3.

Previous study has indicated the oncogenic role of LINC00662 in breast cancer^13,14^. In this study, we will further evaluate its role in docetaxel resistance. Consistent with previous study, we have found higher LINC00662 expression in breast cancer tissues than in non-diseased breast tissues (Fig. 1A). Furthermore, we found that LINC00662 expressed higher in recurrent patients, indicating that LINC00662 might be associated with AC-T resistance. (Fig. 1B) Then, we noticed that m6A levels were enhanced in recurrent TNBC tissues (Fig. 1C). Moreover, ELISA assays showed that METTL3 (Fig. 1D) was upregulated in recurrent TNBC tissues, whereas METTL14 (Fig. 1E), FTO (Fig. 1F) and ALKBH5 (Fig. 1G) were not significantly differentially expressed. More importantly, we found that LINC00662 expression was positively associated with METTL3 expression (Fig. 1H). Therefore, we assumed that METTL3 might regulate LINC00662 to induce docetaxel resistance of breast cancer.

### LINC00662/METTL3 regulated docetaxel resistance of breast cancer cells

**Figure 2:**
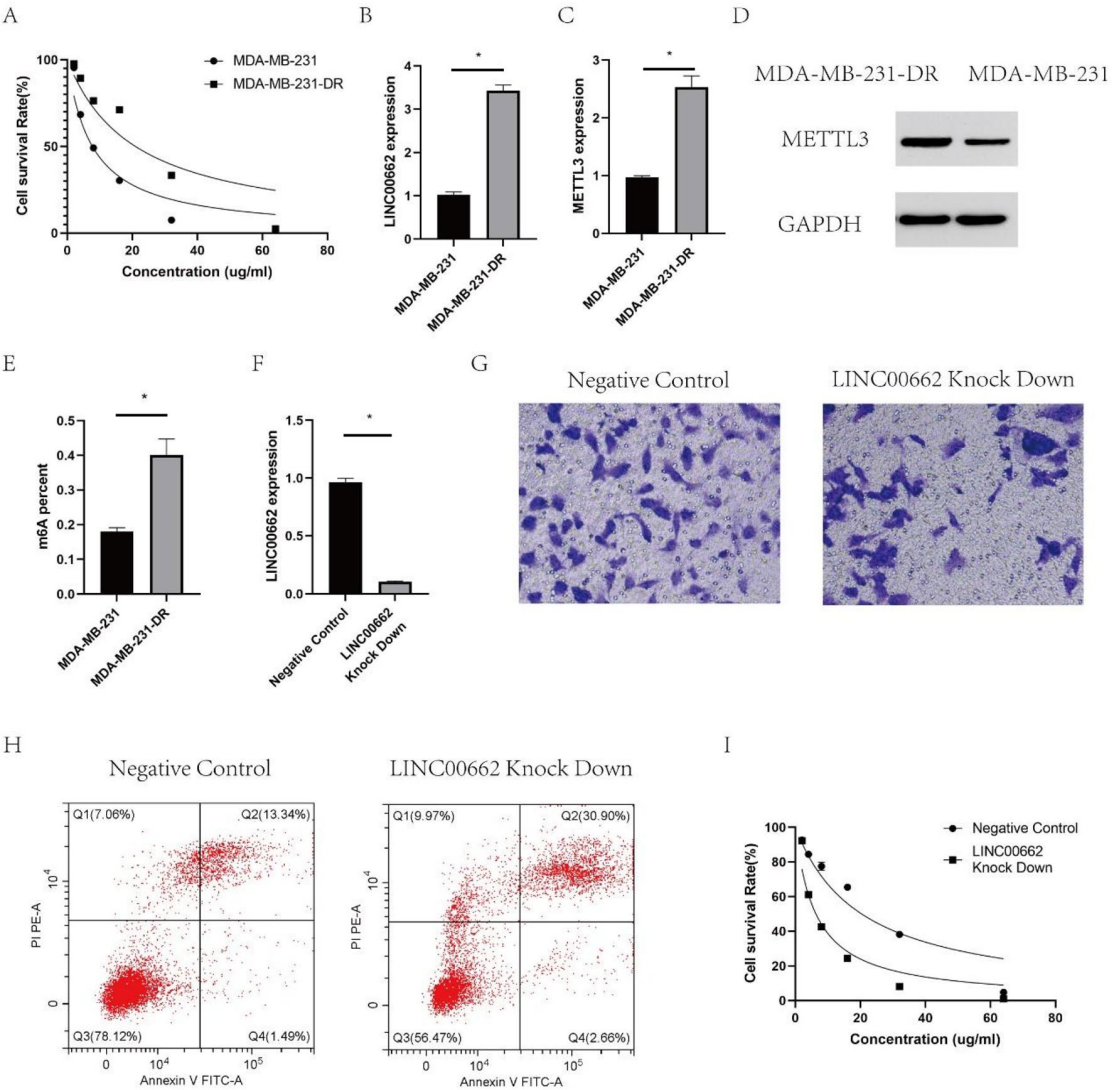
The expression of LINC00662, METTL3 and m6A were higher in docetaxel resistant MDA-MB-231 cells than MDA-MB-231 cells. After knockdown of linc00662 in MDA-MB-231-DR, cell migration decreased and apoptosis rate increased. However, the IC50 of MDA-MB-231-DR decreased after LINC00662 downregulation, suggesting that LINC00662 may be related to docetaxel resistance. (**A**) The IC50 of MDA-MB-231 (7.72 ± 0.89 mg/mL) and MDA-MB-231-DR (20.97 ± 1.32 mg/mL) treated with docetaxel; (**B**) LINC00662 expression in MDA-MB-231-DR compared with MDA-MB-231 by qRT-PCR; (**C**) METTL3 expression in MDA-MB-231-DR compared with MDA-MB-231 by qRT-PCR; (**D**) METTL3 expression in MDA-MB-231-DR compared with MDA-MB-231 by western blot; (**E**) The m6A level in docetaxel resistant MDA-MB-231-DR compared with MDA-MB-231; (**F**) LINC00662 expression in LINC00662 knocked down MDA-MB-231-DR; (**G**) Transwell assay to evaluate the migration ability in LINC00662 knocked down MDA-MB-231-DR; (**H**) Apoptosis results for knocking down LINC00662 in MDA-MB-231-DR; (**I**) The IC50 of LINC00662 knocked down MDA-MB-231-DR (6.32 ± 0.94 mg/mL).

As for in vitro experiments, we have constructed docetaxel resistant MDA-MB-231 cells (MDA-MB-231-DR), the IC50 of which was 20.97 ± 1.32 mg/mL; besides, the IC50 of MDA-MB-231 was 7.72 ± 0.89 mg/mL. (Fig. 2A). We have found enhanced expression of LINC00662 (Fig. 2B) as well as METTL3 by qRT-PCR and western blot (Fig. 2C,D) in MDA-MB-231-DR compared with MDA-MB-231. In addition, m6A level was also enhanced in MDA-MB-231-DR (Fig. 2E). By knocking down LINC00662 in MDA-MB-231-DR (Fig. 2F), we have detected decreased cell migration (Fig. 2G) and increased apoptosis rate (Fig. 2H). Negative control means MDA-MB-231-DR cell lines without knocked down by LINC0662. Further CCK-8 assay showed significantly decreased IC50 of MDA-MB-231-DR after LINC00662 down regulation (6.32 ± 0.94 vs 20.39 ± 3.52, Fig. 2I). Taken together, these data showed that LINC00662 and METTL3 might play important roles in mediating docetaxel resistance.

### METTL3 up-regulated the expression of LINC00662 to induce docetaxel resistance of breast cancer cells

**Figure 3:**
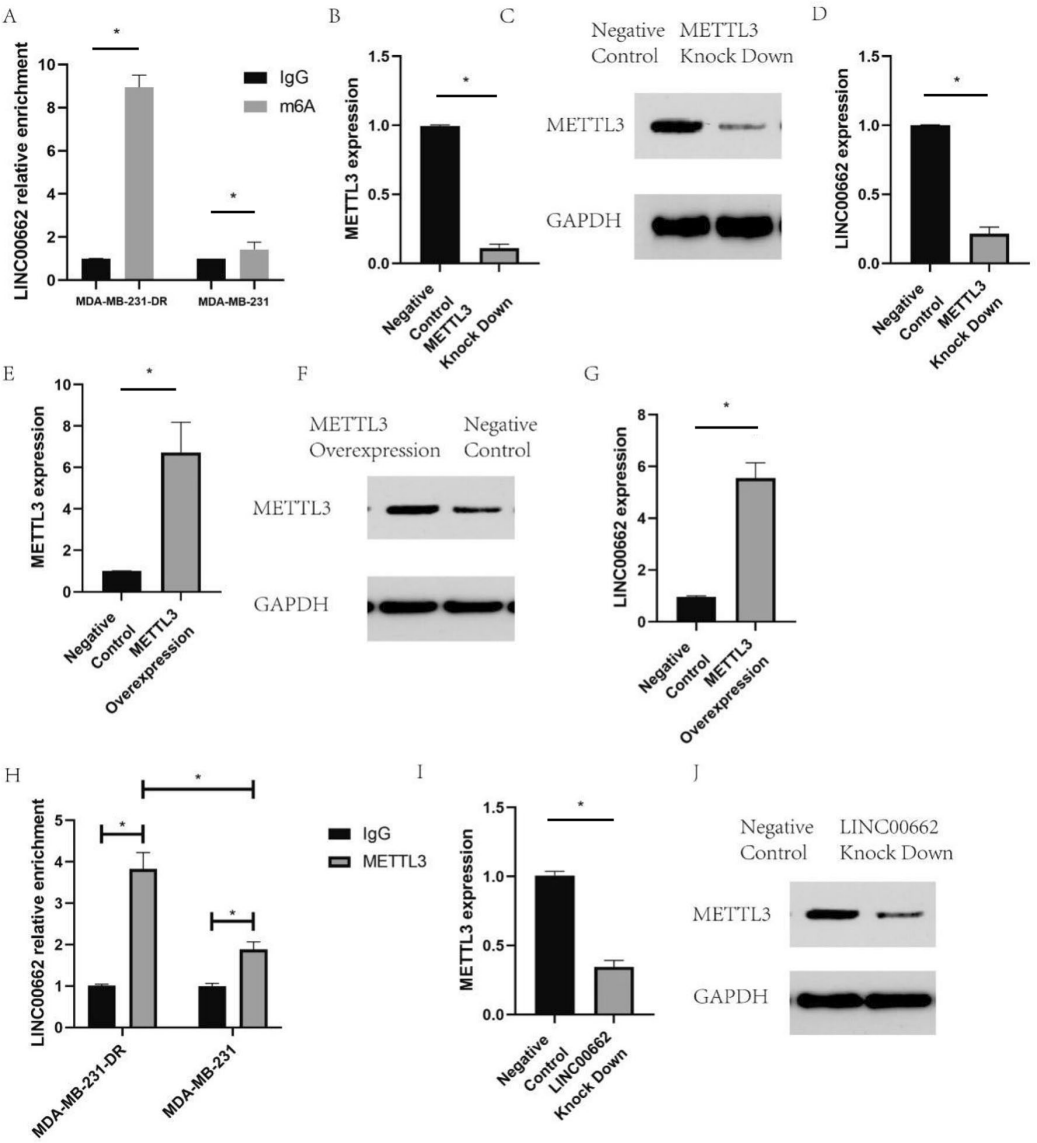
LINC00662 was significantly enriched by m6A antibody and the expression of LINC00662 was downregulated with METTL3 knockdown and upregulated with METTL3 overexpression. RIP assay showed that METTL3 could bind to LINC00662 and LINC00662 knock down also affected METTL3 expression. (**A**) Me-RIP to evaluate the enrichment of LINC00662 by m6A antibody in MDA-MB-231 and MDA-MB-231-DR; (**B**, **C**) METTL3 knock down efficiency in MDA-MB-231-DR by qRT-PCR and western blot; (**D**) LINC00662 expression in METTL3 knocked down MDA-MB-231-DR; (**E**, **F**) METTL3 overexpression efficiency in MDA-MB-231-DR by qRT-PCR and western blot; (**G**) LINC00662 expression in METTL3 overexpressed MDA-MB-231-DR; (**H**) RIP assay to evaluate the interaction between METTL3 and LINC00662. (**I**, **J**) METTL3 expression in LINC00662 knocked down MDA-MB-231-DR by qRT-PCR and western blot.

Me-RIP (Methylated RNA Immunoprecipitation), as a direct method to obtain specific RNA fragments and sequences, has been successfully used to detect the genome-wide modification of RNA methylation. Our study indicated that LINC00662 was significantly enriched by m6A antibody (Fig. 3A). In MDA-MB-231-DR, we have knocked down METTL3 in MDA-MB-231-DR (Fig. 3B,C). Then, we found that METTL3 down regulation led to decreased expression of LINC00662 (Fig. 3D). Besides, METTL3 overexpression (Fig. 3E,F) induced the expression of LINC00662 in MDA-MB-231-DR (Fig. 3G). RIP assay showed that METTL3 could bind to LINC00662 (Fig. 3H). More importantly, LINC00662 knock down also resulted in decreased expression of METTL3 (Fig. 3I,J). Therefore, we hypothesized that a feedback loop existed to mediate the expression of LINC00662 and METTL3.

### LINC00662/miR-186-5p/METTL3 feedback loop regulates docetaxel resistance in breast cancer

**Figure 4:**
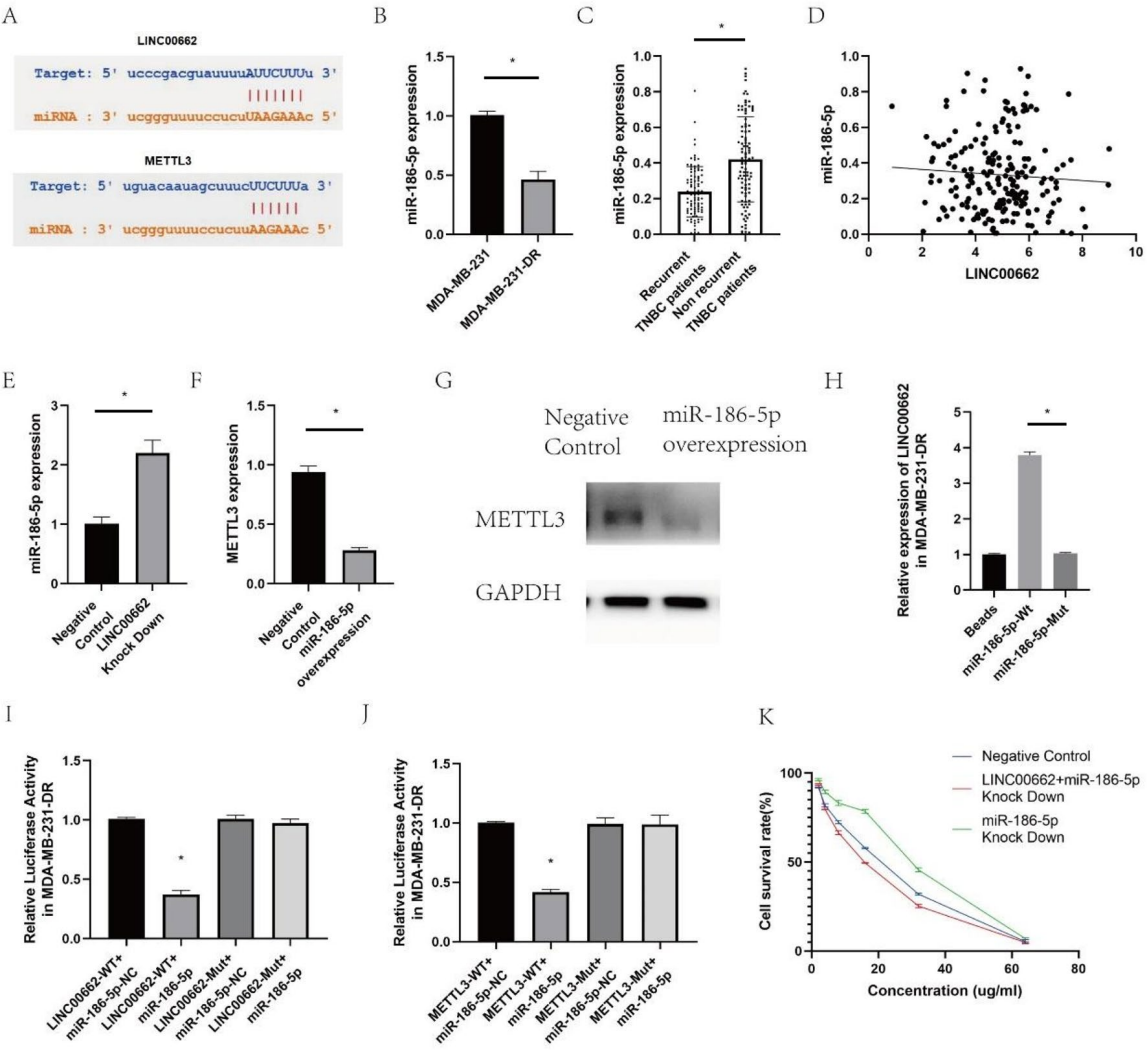
miR-186-5p was predicted to be a potential target of LINC00662 & METTL3. The expression was found to be downregulated in MDA-MB-231-DR and recurrent TNBC tissues. After the study found that linc00662 expression was negatively correlated with mir-186-5p expression in TNBC tissues, it was also found that linc00662 knockdown led to overexpression of mir-186-5p and further reduced mettl3 expression. Besides, LINC00662 was significantly enriched in the miR-186-5p-WT group in RNA pull down, and miR-186-5p could interact with both LINC00662 and METTL3 by dual luciferase reporter assay. (**A**) Starbase to predict the interaction between miR-186-5p & LINC00662 and miR-186-5p & METTL3. (**B**) The expression of miR-186-5p in docetaxel resistant MDA-MB-231-DR compared with MDA-MB-231; (**C**) The expression of miR-186-5p in recurrent and non-recurrent TNBC patients; (**D**) The correlation between miR-186-5p and LINC00662 in TNBC; (**E**) miR-186-5p expression in LINC00662 knocked down MDA-MB-231-DR; (**F**, **G**) METTL3 expression in miR-186-5p overexpressed MDA-MB-231-DR; (**H**) RNA pull down to evaluate the interaction between LINC00662 and miR-186-5p in MDA-MB-231-DR; (**I**) Dual luciferase reporter gene assay to evaluate the interaction of LINC00662 and miR-186-5p in MDA-MB-231-DR; (**J**) Dual luciferase reporter gene assay to evaluate the interaction of METTL3 and miR-186-5p in MDA-MB-231-DR; (**K**) IC50 for LINC00662 and miR-186-5p simultaneously knocked down MDA-MB-231-DR.

Based on Starbase V3.0, we have found that miR-186-5p was the predicted target for both LINC00662 and METTL3 (Fig. 4A). Moreover, we have found decreased expression of miR-186-5p in MDA-MB-231-DR compared with MDA-MB-231 (Fig. 4B). In breast cancer patients, miR-186-5p was downregulated in recurrent TNBC tissues (Fig. 4C); Besides, LINC00662 expression was negatively associated with miR-186-5p expression in TNBC tissues (Fig. 4D).

Furthermore, we noticed that LINC00662 knocking down resulted in overexpression of miR-186-5p (Fig. 4E), besides miR-186-5p overexpression led to decreased expression of METTL3 (Fig. 4F,G). RNA pull down assay also showed that LINC00662 was significantly enriched in the miR-186-5p-WT group, indicating the potential crosstalk between LINC00662 and miR-186-5p (Fig. 4H). Then, dual luciferase reporter gene assay showed that miR-186-5p could interact with both LINC00662 and METTL3 (Fig. 4I,J). In addition, after knocking down LINC00662 and miR-186-5p simultaneously, we found that the IC50 of MDA-MB-231-DR was not significantly changed, whereas miR-186-5p knock down alone significantly increased the IC50 (Fig. 4K).

Taken together, we hypothesized that METTL3 expression was up-regulated to induced docetaxel resistance in breast cancer by activating the expression of LINC00662; moreover, LINC00662 sponged miR-186-5p to further increase the expression of METTL3.

## Discussion

Breast cancer (BC) is one of the most common malignancies in women worldwide, while triple-negative breast cancer (TNBC) is the most aggressive molecular subtype of breast tumors. However, effective therapies are limited by several reasons, such as the complex interaction among different signal pathways and chemoresistance. Emerging evidence indicates that long non-coding RNAs (lncRNAs) are associated with TNBC carcinogenesis. In the current study, we explored the mechanism about METTL3/LINC00662/miR-186-5p feedback loop regulates docetaxel resistance in Triple negative breast cancer. Here, we have have found that METTL3 was overexpressed in docetaxel resistant breast cancer cells; besides, METTL3 could positively mediate docetaxel resistance of TNBC. Furthermore, we have found that LINC00662 could interact with miR-186-5p and miR-186-5p could bind to METTL3 to suppress its expression. Interestingly, METTL3 could induce the expression LINC00662, and thus competing for miR-186-5p binding, and finally leading to METTL3 overexpression to promote docetaxel resistance.

METTL3, a regulator of RNA m6A methylation, has been found to promote chemoresistance in various cancers^7,15^. It has been shown that METTL3 induces chemoresistance by regulating the expression of key genes. For example, METTL3 can regulate MAPK cascades to promote chemoresistance in pancreatic cancer cells^15^. Jin et al. showed that METTL3 plays an essential part as a competing endogenous RNA; METTL3 knockdown decreased the expression of lncRNA MALAT1, thereby suppressing endogenous competition for MALAT1 to bind to miR-646, and finally induced the YAP axis^16^. In this work, we found that METTL3 was also associated with the expression of LINC00662. Experiments indicated the important regulatory role of METTL3 in promoting docetaxel resistance by interfering in the interaction between LINC00662 and miR-186-5p.

LINC00662 has been shown to be oncogenic in various cancers, including colorectal cancer, hepatocellular carcinoma, oral squamous cancer, and prostate cancer^17–20^. In this work, we not only clarified the oncogenic role of LINC00662 in breast cancer through downregulating the expression of miR-186-5p but also demonstrated the important regulatory role of LINC00662/miR-186-5p in regulating docetaxel resistance. Guo et al. showed that LINC00662 was essential in maintaining hypomethylation by reducing SAM and SAH levels via interactions with MAT1A and AHCY, thereby regulating gene expression by altering promoter methylation^17^. Owing to its role in promoting docetaxel resistance, LINC0062 might provide a valuable therapeutic target that could be used to reverse docetaxel resistance.

miR-186-5p has been shown to be a tumor suppressor in osteosarcoma, lung adenocarcinoma, gastric cancer, etc.^21–23^. In non-small-cell lung cancer, miR-186-5p suppresses cisplatin resistance by inhibiting the expression of SIX1^24^. Our results broaden understanding of the role of miR-186-5p in regulating drug resistance. We showed that miR-186-5p functions as a target of LINC00662, decreasing cell viability and promoting apoptosis of docetaxel-resistant breast cancer cells. Dong et al. showed that miR-186-5p interacted with HOXD-AS1 and thereby regulated PIK3R3 activity to suppress epithelial-to-mesenchymal transition (EMT) of ovarian cancer cells^25^. EMT is an important biological process that mediates drug resistance in breast cancer^26^. Moreover, various studies showed that RNA m6A methylation plays an important part in regulating EMT by intervening in the translation of EMT-regulating gene Snail^27^.

In this work, we have found m6A level was associated with docetaxel resistance of TNBC; further experiments have shown that METTL3 might be the key m6A associated gene in regulating docetaxel resistance. In vitro experiments have confirmed that METTL3 could induce docetaxel resistance. Then, we have confirmed the interactions among METTL3, LINC00662 and miR-186-5p. which could shed light on how METTL3 was induced in docetaxel resistanct TNBC. However, more studies are needed to clarify the mechanisms of METTL3 in regulating docetaxel resistance.

## Conclusions

In conclusion, we have presented evidence that RNA m6A plays an important part in regulating docetaxel resistance in breast cancer. Furthermore, METTL3 mainly promotes docetaxel resistance by targeting LINC00662. Finally, LINC00662 directly interacts with miR-186-5p to regulate cell viability and apoptosis in breast cancer. Our results provide insights into possible means of reversing docetaxel resistance through suppressing m6A-related pathways.

## Supplementary Information

Supplementary Legends.

Supplementary Figure S1.

Supplementary Table S1.
