# Supplementary material for: METTL3/LINC00662/miR-186-5p feedback loop regulates docetaxel resistance in triple negative breast cancer
Source: Sci Rep. 2022 Oct 6;12:16715. doi: 10.1038/s41598-022-20477-0 (PMC9537189; doi:10.1038/s41598-022-20477-0)
Supplement: Supplementary file 1 — Supplementary Legends. [file 41598_2022_20477_MOESM1_ESM.pdf]

A. Primary western blot figures for 3C, 3J and 2D.
B. Primary western blot figures for 4G.
C. Primary western blot figures of GAPDH for 3C, 3J and 2D.
D. Primary western blot figures of GAPDH for 4G and related replicates.
E. and F. Primary western blot figures for replicates for 3C, 3J and 2D.
G. and H. Primary western blot figures for replicates of GAPDH for 3C, 3J and 2D.
