# Supplementary figures and images for: METTL3/LINC00662/miR-186-5p feedback loop regulates docetaxel resistance in triple negative breast cancer
Source: Sci Rep. 2022 Oct 6;12:16715. doi: 10.1038/s41598-022-20477-0 (PMC9537189; doi:10.1038/s41598-022-20477-0)

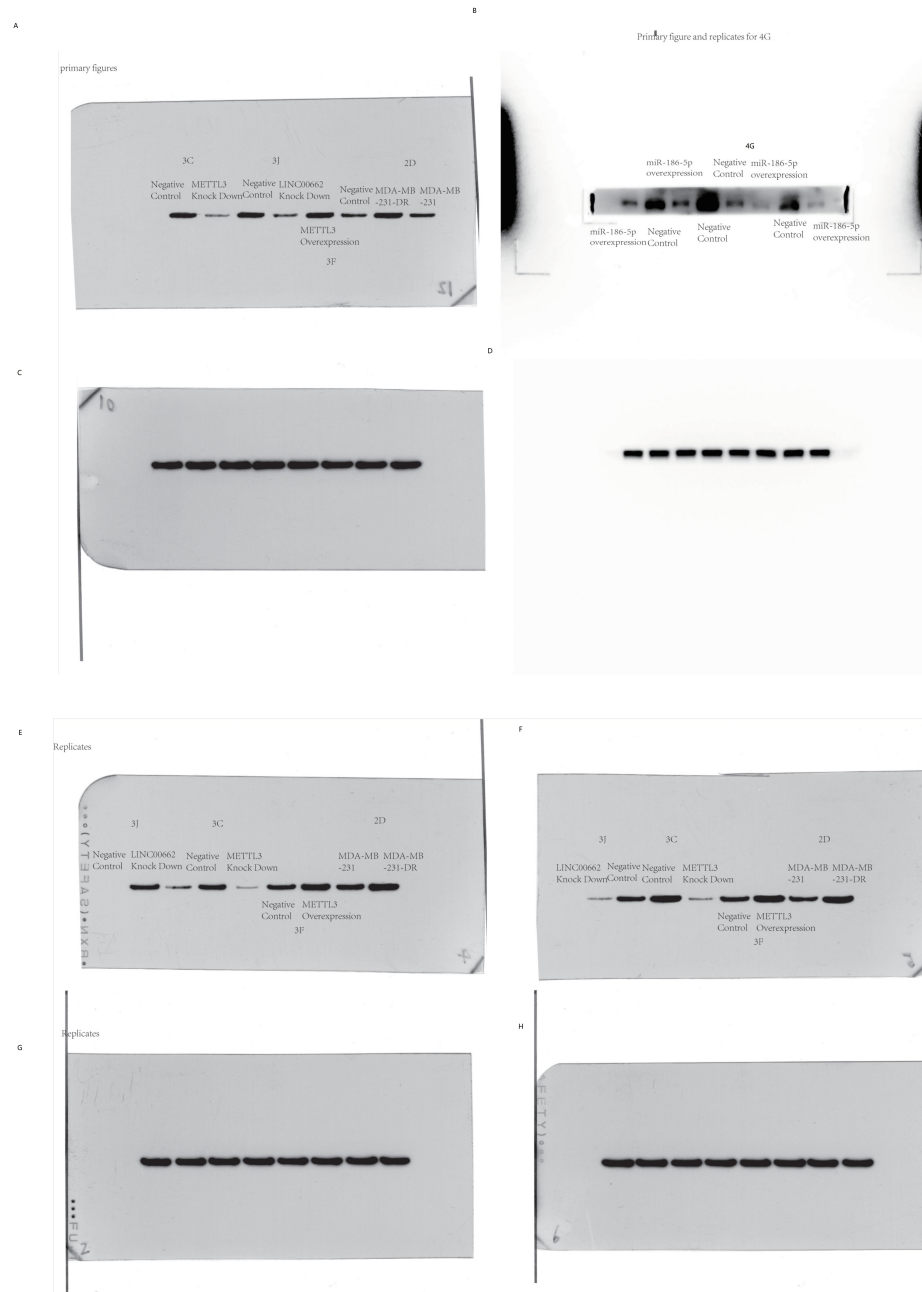

Supplement: Supplementary file 2 — Supplementary Figure S1. [file 41598_2022_20477_MOESM2_ESM.pdf]
