# Supplementary material for: METTL3/LINC00662/miR-186-5p feedback loop regulates docetaxel resistance in triple negative breast cancer
Source: Sci Rep. 2022 Oct 6;12:16715. doi: 10.1038/s41598-022-20477-0 (PMC9537189; doi:10.1038/s41598-022-20477-0)
Supplement: Supplementary file 3 — Supplementary Table S1. [file 41598_2022_20477_MOESM3_ESM.pdf]

Supplementary table 1

Primer sequences for qPCR

**Genes**	**Name**	**Primer sequence (5′-3′)**
LINC00662	qPCR primer F	TGGACATCTGTCTGGAGG
	qPCR primer R	GGCTGAGGCATAAGAATCG
METTL3	qPCR primer F	GTGTCGGAGGTGATTCCAGT
	qPCR primer R	CTGCGCATCTCATCATCTGT
miR-186-5p	qPCR primer F	AAGAATTCTCCTTTTGGGCT
	qPCR primer R	GTGCGTGTCGTGGAGTCG
GAPDH	qPCR primer F	GACAGTCAGCCGCATCTTCT
	qPCR primer R	GCGCCCAATACGACCAAATC
U6	qPCR primer F	TCCCTTCGGGGACATCCG
	qPCR primer R	AATTTTGGACCATTTCTCGATTTGT

Primer sequences for vector construction

**Genes**	**Name**	**Primer sequence (5′-3′)**
LINC00662-WT	Sense	GGGGTACCGTAGTCCGGCCGCCCTGTGA
	Anti-sense	GGAATTCCACCACGACCAAGTGCAATTTATTC
LINC00662-Mut	Sense	UUCUCCGAACGUGUCACGUTT
	Anti-sense	ACGUGACACGUUCGGAGAATT
shRNA-NC	Sense	CACCGTTCTCCGAACGTGTCACGTCAAGAGATTACGTGACACGTTCGGAGAATTTTTG
	Anti-sense	GATCCAAAAAATTCTCCGAACGTGTCACGTAATCTCTTGACGTGACACGTTCGGAGAAC
shRNA-LINC-1	Sense	CACCGCTGCTGCCACTGTAATAAAGTTCAAGAGACTTTATTACAGTGGCAGCAGCTTTTTG
	Anti-sense	GATCCAAAAAGCTGCTGCCACTGTAATAAAGTCTCTTGAACTTTATTACAGTGGCAGCAGC
shRNA-LINC-2	Sense	CACCGCTGCTACTATGCTGAATTTATTCAAGAGATAAATTCAGCATAGTAGCAGCTTTTTG
	Anti-sense	GATCCAAAAAGCTGCTACTATGCTGAATTTATCTCTTGAATAAATTCAGCATAGTAGCAGC
